# Gut microbiota: sculptors of the intestinal stem cell niche in health and inflammatory bowel disease

**DOI:** 10.1080/19490976.2021.1990827

**Published:** 2021-11-07

**Authors:** Manasvini Markandey, Aditya Bajaj, Nicholas Edward Ilott, Saurabh Kedia, Simon Travis, Fiona Powrie, Vineet Ahuja

**Affiliations:** a Department of Gastroenterology and Human Nutrition, All India Institute of Medical Sciences, New Delhi, India; b Kennedy Institute of Rheumatology, University of Oxford, Oxford, UK; c Translational Gastroenterology Unit, NIHR Oxford Biomedical Research Centre, Oxford University Hospitals NHS Foundation Trust, Oxford, UK

**Keywords:** Intestinal epithelial stem cells (ISCs), gut microbiota, crypt specific core microbiota CSCM), paneth cells, enteroendocrine cells, mesenchyme, gut metabolites, inflammatory bowel disease (IBD)

## Abstract

Intestinal epithelium represents a dynamic and diverse cellular system that continuously interacts with gut commensals and external cues. Intestinal stem cells, which lie at the heart of epithelial renewal and turnover, proliferate to maintain a steady stem cell population and differentiate to form functional epithelial cell types. This rather sophisticated assembly-line is maintained by an elaborate micro-environment, sculpted by a myriad of host and gut microbiota-derived signals, forming an intestinal stem cell niche. This complex, yet crucial signaling niche undergoes dynamic changes during homeostasis and chronic intestinal inflammation. Inflammatory bowel disease refers to a chronic inflammatory response toward pathogenic or commensal microbiota, in a genetically susceptible host. Compositional and functional alterations in gut microbiota are pathognomonic of IBD.

The present review highlights the modulatory role of gut microbiota on the intestinal stem cell niche during homeostasis and inflammatory bowel disease. We discuss the mechanisms of direct action of gut commensals (through microbiota-derived or microbiota-influenced metabolites) on ISCs, followed by their effects via other epithelial and immune cell types.

## 1. Introduction

**Figure 1.**
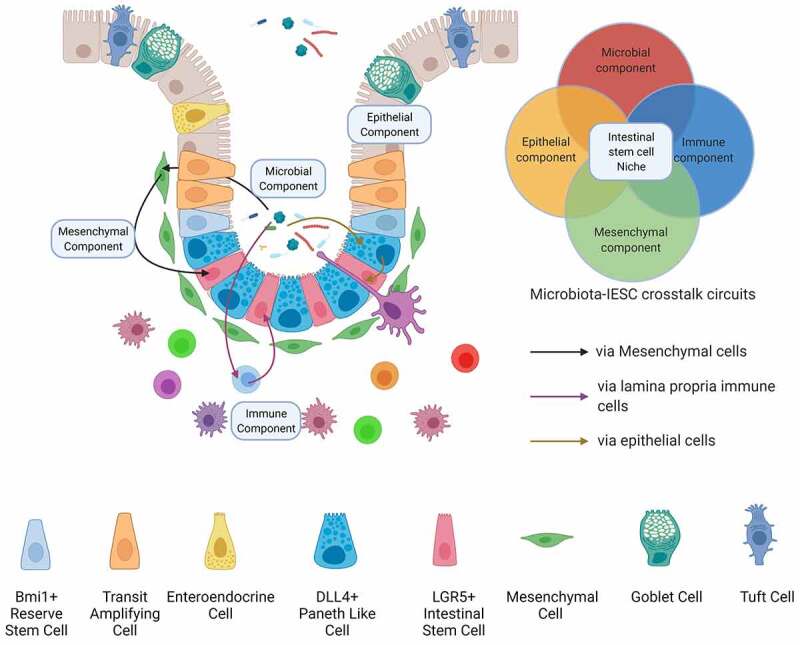
The components of the intestinal stem cell niche. The intestinal stem cell niche, which lies at the heart of gut epithelial renewal and regeneration, constitutes the factors contributed by four intestinal cellular compartments – the gut microbiota, the epithelium, immune cells of the underlying lamina propria and the mesenchymal cells. The gut microbiota regulates ISC physiology either directly or through other niche components, shaping the epithelial renewal during homeostasis and post-injury regeneration

The intestine represents a dynamic environment that is continuously exposed to physiological, pathogenic, and environmental stimuli. To combat such harsh conditions, intestinal epithelium acts as a robust barrier that protects the underlying tissues from potential injuries and infections. Composed of an array of functionally differentiated cells, the epithelium carpets the entire span of the intestine, folding back and forth upon itself to form alternating villi and crypts. The base of these intestinal crypts is inhabited by the intestinal stem cells (ISCs), which form the epicenter of the gut regenerative machinery, dividing continually to replenish their own population as well to generate the differentiated epithelial subtypes. These multipotent cells are identified by a marker gene – leucine-rich repeat G protein coupled receptor (LGR5), and when isolated, can solely give rise to complete 3D mini-intestines in vitro – the organoids.^1,2^ The ISCs divide to give rise to transit amplifying cells, which further generate various differentiated epithelial subpopulations, replenishing the differentiated cells lost to programmed-cell death (anoikis) in the gut lumen. Cues for the sustenance of the homeostatic proliferation-to-differentiation ratio of stem cells are produced by various cellular entities in the vicinity of the crypts, including the gut microbes. The microenvironment encapsulating these stem cells, along with nourishing factors, their producers and mediators, is called the stem cell niche. The ISC niche can be envisioned as a four-component system, composed of the epithelial, mesenchymal, immune and the microbial components (Figure 1).

**Figure 2.**
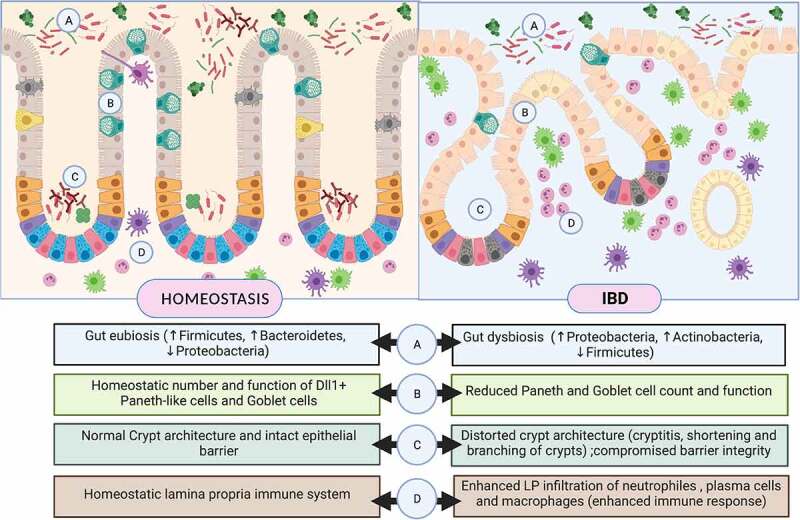
The intestinal stem cell niche along colon in homeostasis (left panel) and Inflammatory Bowel Disease (right panel). Intestinal stem cell niche comprises of intestinal stem cells and Paneth-like population of Dll4+/Reg4+ Deep Crypt Cells (DCS) located along the crypt base. ISCs undergoes continual renewal and differentiation into distinct secretory and absorptive epithelial cell types. Other sub-niches such as the gut microbiota and underlying layer of immune cells, contribute to the myriad of signals which drive this complex and dynamic process of epithelial renewal and differentiation. IBD-associated alterations in ISC niche and the associated factors have been highlighted along the lower panel

Inflammatory bowel disease, a debilitating chronic inflammatory disorder, is characterized by a severely dysfunctional epithelium, involving drastic reprogramming of ISC niche structure and function. Histologically, IBD is characterized by distortions in crypt architecture, such as shortening and branching of the crypts, cryptitis, or crypt abscesses. The two forms of IBD, namely UC and CD, are characterized by reduced numbers and altered functions of Goblet Cells (GC) and Paneth Cells (PC) respectively, which are involved in the maintenance of the mucosal barrier, eliciting antimicrobial responses and activating immune cells toward the translocating antigens. Alterations in the differentiation of functional Paneth cells and goblet cells from ISCs can be attributed to an altered IBD-associated re-wiring of epithelial progenitors (Figure 2).

Gut commensals form an indispensable niche factor that apart from forging direct interactions with the ISCs, synergizes signaling cascades with other epithelial cell types in the ISC niche. The gut microbiota is a complex consortium of commensal microorganisms, strategically lodged in diverse functional niches along the intestine. Not only these microbes have marked their presence in the intestinal stem cell niche,^3,4^ but also the products of their metabolism cast out multitude of direct and indirect (involving other ISC niche cell types) interactions with ISCs, thereby modulating epithelial function, restitution and repair. The present review attempts to accentuates the dialog at the ISC–IBD–microbiota trijunction during homeostasis and chronic intestinal inflammation. Beginning with the IBD-specific rewiring of the gut regenerative machinery, we shall delve into a myriad of direct and indirect interactions between the ISC niche and its resident flora shaping the niche function.

## 2. Inflammatory Bowel Disease – specific ISC Rewiring

The intestinal epithelial barrier forms the most significant interface between exogenous antigens and the mucosal immune system in the human body. Intestinal stem cells maintain this dynamic layer by ensuring turnover and restitution. Inflammatory bowel disease, encompassing ulcerative colitis (UC) and Crohn’s disease (CD), is the manifestation of a prolonged, uncontrolled immune response induced by environmental cues, toward pathogenic or commensal microbiota, in a genetically vulnerable host.^5^ Even though the temporal order of these events in the pathogenesis of IBD remains ambiguous, it cannot be denied that a dysfunctional intestinal epithelial barrier, and translocation of gut bacteria, or their antigens, is an indispensable trigger for progression to chronic inflammation. The IBD-associated epithelial barrier breach is marked by increased epithelial cell death,^6^ reduced expression of tight junction proteins^7^ and deficient antimicrobial response elicited by malfunctioning Paneth and goblet cells.^8–13^ Consequent gaps in the barrier allow percolation of luminal contents into the lamina propria, thereby stimulating resident and circulating immune cells. The mucosal healing process that follows is propelled by the regenerative capacity of the LGR5+ ISCs.

The two forms of IBD, namely UC and CD, are characterized by reduced numbers and altered functions of Goblet Cells (GC) and Paneth Cells (PC), respectively, which are involved in the maintenance of the mucosal barrier, eliciting antimicrobial responses and activating immune cells toward the translocating antigens.^8–10^ Alteration in the differentiation of functional Paneth cells and goblet cells from ISCs can be attributed to an altered IBD-associated re-wiring of epithelial progenitors (Figure 2).^11^ This phenomenon can be witnessed in gut organoid cultures derived from patients with CD. The enteroids established from biopsies taken from active lesions show altered profiles of ISC-marker genes (such as OLFM4 and SLC12A2) and a higher organoid reformation ability when compared with organoids derived from mucosa under remission.^12^

Factors driving this ISC reprogramming during chronic inflammation include epigenetic modifiers, transcriptional factors, coactivators, and cellular enzymes. For instance, a key ISC-specific transcription factor Tcf-4, responsible for differentiation of Paneth cells, is found to be downregulated in mucosa of patients with ileal CD, and correlates with compromised alpha defensin production.^13^ On the epigenetic front, an ISC-predominant histone methyltransferase, SETDB1 has been underlined for its role in epithelial homeostasis. SETDB1 supports transcriptional repression, through trimethylation of H3K9 residue. ISC-specific SETDB1 knockout mice show villus atrophy, crypt disfigurement, enhanced crypt proliferative compartment and reduced goblet and Paneth cell numbers in the gut epithelium, eventually causing the mice to develop a colitis-associated-cancer phenotype.^14^ Interestingly, recent studies have reported the downregulation of SETDB1 enzyme, as well as the over-representation of mis-sense SETDB1 variants in patients with IBD, strengthening its involvement in IBD-associated epithelial dysmorphia.^15^ Other such modifiers include Sirtuin2 (SIRT2), Steroid receptor coactivator (SRC-3) and Liver Receptor Homolog-1 (LRH-1). These modifiers have been found to be reduced in intestinal tissues of patients with IBD and have been implicated in regulating ISC proliferation (Wnt -β-catenin and Notch) and differentiation-specific signaling in IBD.^16–18^ This ‘IBD-specific ISC-rewiring’ is said to contribute to a cellular inflammatory memory/imprint, where dysfunctional epithelial cells borne from imprinted ISCs release higher levels of immune cytokines and nitric oxide (NO), contributing to hyperactivation of the mucosal immune system. This could be the cause of relapse or flare of disease after healing and may participate in the progression of colitis-associated cancer, due to the prolonged inflammation.^19^ Recent studies underlining the rewiring of ISC proliferation and differentiation, the underlying mechanism(s) and their implications in IBD have been highlighted in Table 1.

**Table 1.** Summary of studies describing ISC-specific modulatory effectors and their association with Human IBD and/or DSS-colitis

**ISC-modulating effector/factor**	**ISC modulation and/or underlying mechanism(s)**	**Association with Human IBD and/or DSS-colitis**
Sirtuin 2 (SIRT2)^16^	SIRT2- deficient mice show reduced ileal enterocyte and GC differentiation and increased IEC proliferation. SIRT2 inhibits Wnt/β-catenin signaling.	SIRT2 expression markedly reduced in IBD.
SET Domain Bifurcated Histone Lysine Methyltransferase 1 (SETDB1)^14,15^	IEC-specific Setdb1 deletion show reduced GCs and EECs in SI, along with mis-positioning of PCs.	Reduced SETDB1 expression in human IBD and in DSS mice model of colitis; Over-representation of rare missense variants of SETDB1 in human IBD.
Liver Receptor Homolog-1 (LRH-1)^18^	LRH-1 KO mice show reduced Notch signaling, increased crypt cell death, distortion in epithelial cellular composition. LRH-1 potentiates Wnt/β-catenin signaling and epithelial renewal.	GWAS have implicated LRH-1 in IBD. Human LRH-1 (hLRH-1) rescues epithelial integrity and when overexpressed, mitigates inflammatory damage in murine and human intestinal organoids.
CD47^20^	CD47 inhibits IEC proliferation and migration by suppressing four Yamanaka transcriptional factors (OSKM – Oct4, Sox2, Klf4 and c-Myc)	CD47 expression enhances in UC and CD as well as in mouse experimental colitis models.
X-box–binding protein 1 (XBP1)^21^	Hypomorphic variants of XBP1 drive Ire1α–mediated increase in Lgr5+ and Olfm4+ ISCs and a Stat3-dependent increase in the proliferative output of transit-amplifying cells.	Hypomorphic variants of ER stress response mediators, such as XBP1, confer genetic risk for IBD

GCs, Goblet cells; IECs, Intestinal epithelial cells; EECs, Enteroendocrine cells; SI, Small Intestine; PCs, Paneth cells; DSS, Dextran Sodium Sulfate; KO mice, Knockout mice

It is noteworthy that intestinal stem cells are epigenetically imprinted with a functional memory of the specific intestinal site from where they derive. To draw an analogy, think of salmon being able to find their way back to the river of their birth after years at sea. Consequently, the organoids arising from these stem cells display conservation of site-specific gene expression patterns. In light of these observations, data from different segment-specific organoid experiments cannot be extrapolated without further experimentation. Hence, we state the location of observation for each study in this review.

## 3. Direct effects of gut microbiota- derived metabolites on ISCs

Advances in bio-imaging and molecular characterization of microbes have equipped us to explore microbial communities in distinct intestinal niches, such as mucosa-associated microbiota (MAM) and crypt-associated microbiota. Laser capture microdissection (LCM), coupled with 16s rRNA qPCR, enables crypt-autochthonous bacterial populations to be studied. Rowan et al. report the presence of commensal bacteria within the crypt-associated mucus gel in health and colitis, with bacterial counts being significantly reduced in patients with acute ulcerative colitis.^22^ A ‘crypt-specific core microbiota’ (CSCM), seated deep at the base of cecal and colonic crypts, was established by Pedron et al. This population is distinct from the MAM and consists of aerobic bacteria like *Acinetobacter, Delftia*, and *Stenotrophomonas sp*. in mice. Human colonic crypts are colonized predominantly by *Firmicutes*, with small proportions of the bacteria characterized in murine crypts.^3,4^

The past decade has witnessed a surge of elegantly designed studies investigating contributions of the gut microbiota in IBD. Accumulating evidence, such as – alleviation of IBD-associated symptoms in antibiotic or prebiotic-treated patients,^23^ IBD-susceptibility-loci being involved in pathways for microbial sensing,^24^ occurrence of impaired mucosal healing and chronic inflammation in absence of commensal microbiota^25,26^ or an inability to induce colitis in germ-free animals – all together promote the hypothesis that gut microbiota is a causative factor in IBD progression. Within the ISC niche, the gut microbiota can interact with ISCs and their niche members in a variety of manners, thereby modulating epithelial function, restitution and repair. Hence, it is relevant to discuss the direct and indirect interactions that gut microbiota can forge with the ISCs during homeostasis and chronic intestinal inflammation. The discussion shall include the crosstalk of gut microbiota, with other niche members as well, that are known to regulate the balance between ISCs proliferation and differentiation.

One of the earliest reports on the role of microflora in the maintenance of crypt structure came from the 1960s, which reported that germ-free or antibiotic-treated rodents exhibited reduced villus height and crypt depth in the jejunum and ileum, reduced mucosal surface area and lower mitotic indices when compared to conventionally raised animals.^27,28^ Studies on humanized microbiome gnotobiotic mouse models have described how gut microbiota transfer from preterm infants who undergo normal or poor weight gain respectively, can differentially shape the crypt-villus architecture, cell proliferation and numbers of Paneth cells and goblet cells.^29^

**Figure 3.**
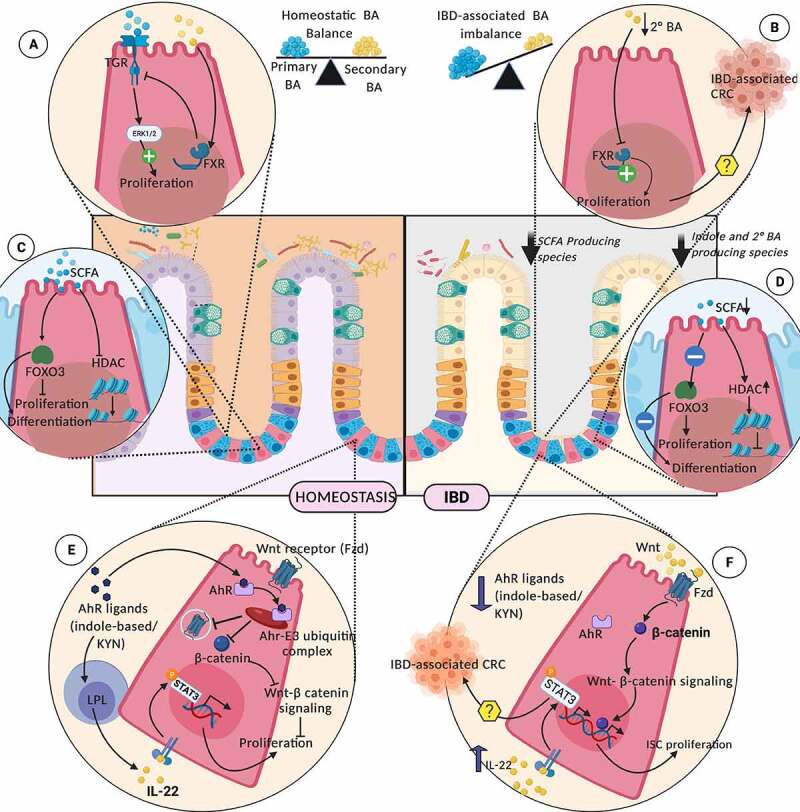
Interaction of Gut Microbiota-derived or influenced metabolites on ISC niche during homeostasis (left panel) and IBD (right panel); Homeostatic levels of primary and secondary bile acid pools, through membrane-bound TGR and cytosolic FXR, is critical for maintaining ISC proliferation (a). Deterioration of microbiota producing the secondary BAs during IBD, promotes uncontrolled ISC proliferation (b). SCFAs modulate ISC physiology through transcriptional and epigenetic regulation (c). IBD- associated dwindling levels of SCFAs, disturbs these regulatory pathways (d). Microbiota-derived AhR ligands checks the ISC proliferation through degradation of β-catenin, by ubiquitination and preventing the cell surface expression of Wnt ligands on ISCs (e) The loss of these regulatory pathways due to IBD-associated degradation of gut indole pool, may drive uncontrolled ISC proliferation (f). TRG (G-protein coupled bile acid receptor), BA (Bile acid), FXR (Farnesoid X Receptor), SCFA (Short-chain fatty acids), HDACs (Histone deacetylases), KYN (Kynurenine), AhR (Aryl-hydrocarbon receptor), Fzd (Frizzled)

Mechanisms of how gut microbiota-derived metabolites shape ISC niche signaling is summarized in Figure 3. These interactions are discussed below.

### 3.1. Bile acids

Bile acids are synthesized in the liver, followed by their conjugation with glycine or taurine and are excreted into the bile canaliculi, from where they are released in the small intestine to facilitate fat emulsification and absorption of fats and cholesterol. These are largely reabsorbed in the terminal ileum and only 5% of these primary bile acids reach the colon, where they are transformed by the resident microflora to secondary bile acids (lithocholic acid and deoxycholic acid).

Colonic deconjugation of primary bile acids is carried out by bile salt hydrolases, which are widely expressed by gram-positive and some gram-negative bacteria, principally by *C. scindens, C. hiranonis, C. hylemonae* (Clostridium cluster XVIa), and *C. sordelli* (Clostridium cluster XI), which are capable of producing secondary bile acids.^30^ Hence, colonic microbiota composition defines the colonic bile acid signature, which in turn shapes the hydrophobicity/hydrophilicity ratios of the colonic bile acid pool.^31,32^ Bile acid pools have been found to be altered in IBD, with increased fecal conjugated primary bile acids and reduced unconjugated secondary bile acids. This IBD-associated alterations in the bile acid pool can be attributed to a reduction in bacterial species (members of *Clostridia*) due to dysbiosis.

Bile acids have been widely implicated as regulators of epithelial cell physiology and pathophysiology. They are sensed by a G-protein-coupled bile acid receptor GPBAR-1 (TGR-5) and epidermal growth factor receptor (EGFr), or by nuclear receptors farnesoid X receptor (FXR), pregnane X receptor (PXR) and vitamin D receptor (VDR). The impact of bile acids on intestinal homeostasis in the ISC niche has recently been elucidated by Sorrentino et al. Apart from manifesting their anti-inflammatory effects through membrane-bound GPBAR1 (TGR5),^33^ these BAs also regulate the ISC number and function through the receptor. The BA-TGR5 interaction drives the SRC/yes-associated protein (YAP) regenerative cascade, thereby supporting ISC renewal, both during homeostasis and damage-induced epithelial regeneration, as well as promoting ISC differentiation to enteroendocrine L-cells.^34^

Bile acids regulate the intestinal cell proliferation and turnover through their interaction with EGFR and FXR. Secondary bile acids such as lithocholic acid (LCA) and deoxycholic acid (DCA) have been implicated in contradictory roles by signaling through FXR. FXR antagonist activity of secondary BAs has been demonstrated, indicating that these compounds are positive effectors of colorectal cancer progression.^35,36^ However, taurine-*conjugated* cholic acid-induced proliferation has been reported through phosphorylation of Src, EGFR, and ERK 1/2, while *unconjugated* BAs act through FXR-dependent mechanisms, inactivating the EGFR, Src, and ERK proteins, thereby inhibiting proliferation.^37^ These contradictory results can be explained by differences in the concentrations of BA to which cells were exposed in the studies. Physiological levels of secondary BA in ISCs support signaling as FXR agonists, keeping a check on Wnt-β Catenin signaling and ISC proliferation. In contrast, lower secondary BA levels (as seen in IBD), function as toxic FXR antagonists, mediating uncontrolled Wnt-β catenin signaling and possibly driving the ISCs to carcinogenesis (Figure 3a and Figure 3b). If the microbiota regulates the concentration of secondary BA, the relevance is apparent.

### 3.2. Short-chain fatty acids (SCFAs)

SCFAs are abundant luminal metabolites produced upon microbiota-driven anaerobic fermentation of partially or completely indigestible dietary polysaccharides. Apart from their immunomodulatory roles in the gut, SCFAs support colonocyte growth and metabolism by acting as a carbon source. SCFAs have also been shown to have a profound impact on the proliferation and differentiation programming of the ISC compartment.^38^ Oral supplementation of SCFAs has been reported to restore proliferation and differentiation of crypt epithelium, through GPCR-mediated activation of MEK-ERK signaling.^39^ Studies using exposure of intestinal organoid cultures to SCFAs revealed anti-proliferative and pro-differentiative effects of SCFAs on ISCs, through modulation of transcription factor Foxo3^40^ (Figure 3c and Figure 3d). This contrasting influence of SCFAs on ISCs and colonocytes has been termed the ‘butyrate paradox’.^41,42^ Butyrate exerts this differential effect on ISCs by acting as a potent inhibitor of histone deacetylases (HDAC), resulting in chromatin remodeling, altered gene expression and reduced proliferation. In contrast, colonocytes are resistant to SCFA-mediated HDAC inhibition. This could be explained by the fact that both colonic cell types are metabolically distinct: while colonocytes rely on fatty acid oxidation and the TCA cycle to meet their energy needs, colonic ISCs rely on glycolytic pathways, leading to accumulation of SCFAs that bring about their anti-proliferative effects. This differential effect of microbiota-derived SCFAs highlights the significance of crypt architecture and colonocytes metabolizing these SCFAs to protect the underlying stem cell population by maintaining a butyrate gradient along the crypt axis. Diverse roles of SCFAs in molding the ISC niche make it evident that any alteration in the metabolism or production of SCFAs will result in dysregulated ISC physiology. IBD is marked by impaired butyrate oxidation in the colonic mucosa, reduced fecal SCFA levels, and functional or compositional alterations in gut microbiota. IBD associated gut dysbiosis shows a marked reduction in the ability of resident flora to metabolize dietary fiber to SCFAs, with depletion of SCFA producers like *Roseburia hominis* and *Faecalibacterium prausnitzii*.^43–45^ As inflammation ensues, the host epithelium becomes less responsive to the available butyrate due to reduced expression of butyrate transporters, receptors and SCFA metabolizing enzymes. Inflammation-induced ISC-specific alterations in SCFA metabolism remain elusive.

### 3.3. Tryptophan-based metabolites

Tryptophan, an essential aromatic amino acid, is metabolized by either host cell-driven kynurenine and serotonin pathways, or a gut microbiota-mediated indole pathway. The latter forms indole-based metabolites that act as ligands for Aryl hydrocarbon Receptor (AhR) on host cells, which shape epithelial cell physiology, barrier integrity, local and distant immune responses. Alterations in tryptophan metabolism have been widely implicated in IBD.^46,47^ Studies have shown a negative correlation between disease activity and indole-based metabolites, serum tryptophan levels, and AhR expression in inflamed mucosa. This altered Trp metabolism in IBD can be attributed to the IBD-dysbiosis. CARD9 knockout mice exhibit altered tryptophan metabolism of gut microbiota, accounting for their inability to produce AhR ligands, thereby mediating colitis in Card9(-/-) mice. AhR activating strains of *Lactobacillus* and AhR agonists were found to ameliorate the DSS-induced colitis in these knockouts.^48^

AhRs are known to be highly expressed in mouse Lgr5+ stem cells and their activation by their synthetic ligands, such as 6-formylindolo[3,2-b]carbazole (FICZ) and 2,3,7,8-tetrachlorodibenzo-*p*-dioxin (TCDD), inhibit organoid development from mice ISCs. These AhR ligands can suppress β-catenin levels, thereby inhibiting Wnt signaling and reducing the proliferation of crypt epithelial cells.^49^ Ablation of epithelial cell-specific AhR has also been reported to drive uncontrolled ISC proliferation and malignant transformation. AhR signaling is a negative regulator of Wnt- β-catenin pathway in ISCs.^50^ Although no study has directly reported gut stem cell modulatory activity of these microbiota-derived indoles, the phytochemical and natural AhR ligand Indole 3-carbinol, has a role in stimulating ISCs to undergo goblet cell differentiation by inhibiting Notch1 signaling pathways. Nevertheless, microbially produced indoles have not yet been studied, since the gut stem cell-modulatory potential of indole-based metabolites has been the subject of study.^51^

The kynurenine production pathway, found in both epithelial and immune cells, involves the enzyme indoleamine 2,3-dioxygenase (IDO) 1, which is stimulated by the gut microbiota. IDO1 expression in the intestinal epithelium promotes secretory cell differentiation in ilea of healthy individuals and patients with CD.^52^ Metabolic products of the kynurenine pathway such as Kynurenine (KYN), Kynurenic acid and melatonin, interact with AhR, activating its E3 ubiquitin ligase activity. This AhR E3 ubiquitin activity mediates degradation of positive effectors of cell proliferation including β-catenin, Akt, ERK, and p38 kinases, thereby impeding Wnt and Notch signaling in the niche cells.^53^ Apart from its anti-proliferative effects, KYN has also been reported to induce differentiation of colon cancer cells to goblet cell lineage. Hence, metabolites of tryptophan metabolism establish beyond doubt that microbial metabolism and its products can regulate the ISC niche during homeostasis and IBD (Figure 3e and figure 3f).

Another mechanism underlining the protective role of microbiota toward ISCs in IBD involves the activation of lamina propria lymphocytes, through Ahr ligands. Described in DSS colitis model, microbially derived indole 3-aldehyde, via Ahr signaling in LPLs, mediates IL-22 release in the ISC niche. IL-22 then stimulates the ISC proliferation in a STAT3- dependent manner.^54^

### 3.4. Bacterial cell wall-derived antigens

Bacterial cell components such as cell wall molecules (lipoteichoic acid, peptidoglycan, lipopolysaccharides, lipid A and muramyl dipeptide) and flagellin are key modulators of gut epithelial proliferation and differentiation.

#### 3.4.1. Muramyl dipeptide (MDP)

A peptidoglycan motif common to all bacteria, is an agonist of NOD2. ISCs express these cytoplasmic pattern-recognition receptors and MDP administration has been reported to induce a higher yield of small intestinal organoids. Levy et al. reported the cytoprotective effects of microbiota-derived MDP, mediated by clearance of excessive reactive oxygen species in the ISCs, thereby maintaining epithelial regeneration. This ROS clearance upon MDP stimulation depends on the mitophagy-mediated elimination of damaged mitochondria through ATG16L1 and NOD2, and interestingly, these genetic loci have been widely implicated as IBD- susceptibility loci.^55,56^ The role of MDP in colitis was further strengthened by a recent report highlighting that the Gram-positive bacteria promote colitis in DSS model via upregulation of MDP-NOD2 pathway and paeoniflorin, a selective MDP inhibitor, is able to decrease the infiltration of these bacteria in intestine and alleviate mice colitis by inhibiting the MDP-NOD2 pathway.^57^

#### 3.4.2. Flagellin

Exposure to the ISC niche drives a reduction in expression of transcription factors, driving the differentiation of progenitors toward absorptive lineages (Hes1) and goblet cells (Hath1 and KLF4).^58^ Recently, an interesting study by Post et al. demonstrated another cell-intrinsic route via which flagellin regulates ISC proliferation. Flagellin–TLR5 interaction on ISC signals upregulation of ISC specific NOX1 expression, leading to higher ROS production, which potentiates activation of EGFR and enhanced proliferation of ISCs.^59^ NOX1 has also been reported to support proliferation of intestinal crypt cells in response to mucosal injury driven by bacterial stimulus.^60^ Interestingly, NOX1 loss of function variants have been shown to be prevalent in patients with IBD.^61,62^ This lack of functional NOX1 gene could explain the inability of ISCs to adapt and proliferate in response to microbial dysbiosis, due a dysfunctional Flagellin-TLR-NOX1 axis. Additionally, the beneficial role of flagellin is corroborated by a prior study wherein flagellin was shown to exhibit a cytoprotective role in small intestinal crypt epithelial cells against radiation exposure.^63^

#### 3.4.3. Bacterial lipopolysaccharides (LPS)

Exert its immunomodulatory effects through interaction with TLR4 on cell surfaces, whereby it activates multiple inflammatory signaling pathways, through activation of NFkB.^64^ LPS has been implicated in IBD as a perpetuator of inflammation as has been highlighted in patients with CD having high serum LPS levels, which correlates strongly with disease severity.^65^

LPS mediates their ISC-modulatory effects via TLR4 on the ISC surface and results in enhanced apoptosis of crypt epithelial cells. The anti-proliferative effects of LPS have been demonstrated in small intestinal organoid models and in ISC-specific TLR4 knockout mice. In the latter, LPS-mediated anti-proliferative effects were completely masked only in crypts borne from TLR4-deficient ISCs, but showed efficient apoptotic cell death in TLR-expressing crypts. Mechanistically, LPS-induced apoptosis was due to p53-upregulated modulator of apoptosis (PUMA) upregulation, through TIR domain-containing adapter-inducing interferon-β (TRIF) in a Myd88 and TNF-dependent manner.^66^ Further strengthening the role of LPS-mediated TLR4 activation in ISCs, the effect of CSCM-derived LPS on colonic epithelium proliferation-to-differentiation balance has been demonstrated. LPS has dual effects on the ISC compartment, first by inhibiting cell proliferation necroptosis of stem cells or transit-amplifying cells, and second enhancing cell differentiation, especially of the goblet cells.^67^

## 4. Modulation of ISCs by Gut Microbiota via the Epithelial Route

### 4.1. Through Paneth Cells

Paneth cells are localized to the base of the crypts and lie adjacent to the intestinal epithelial stem cells. They can sense microbial cues via TLR-Myd88 mediated pathways, in turn mounting antimicrobial defense by releasing anti-microbial peptides, lysozyme, and phospholipase A. However, it is now known that another way in which microbial sensing by Paneth cells can protect epithelial homeostasis is by the release of Wnt ligands and stem cell growth factors. Lactic acid, produced by gut bacteria such as *Lactobacillus* and *Bifidobacterium sp*., is sensed by G-protein-coupled receptors on Paneth cells, triggering the release of Wnt3 in the crypt environment. The consequent ISC proliferation and epithelial regeneration protects mice exposed to radiation- and chemotherapy-induced intestinal damage.^68^ Incidentally, lactic acid producing bacteria are well known as probiotic therapeutics for alleviating symptoms of IBD.^69^ Considering the role of lactic acid in maintenance of epithelial proliferation, the beneficial role of these probiotics in IBD, might be attributed to their ability to stimulate Wnt release from Paneth cells. In colonic crypts, the absence of Paneth-derived proliferative signals may be compensated by Wnt-producing Paneth cell-like populations, found to be inter-digitated between ISCs.^70,71^

**Figure 4.**
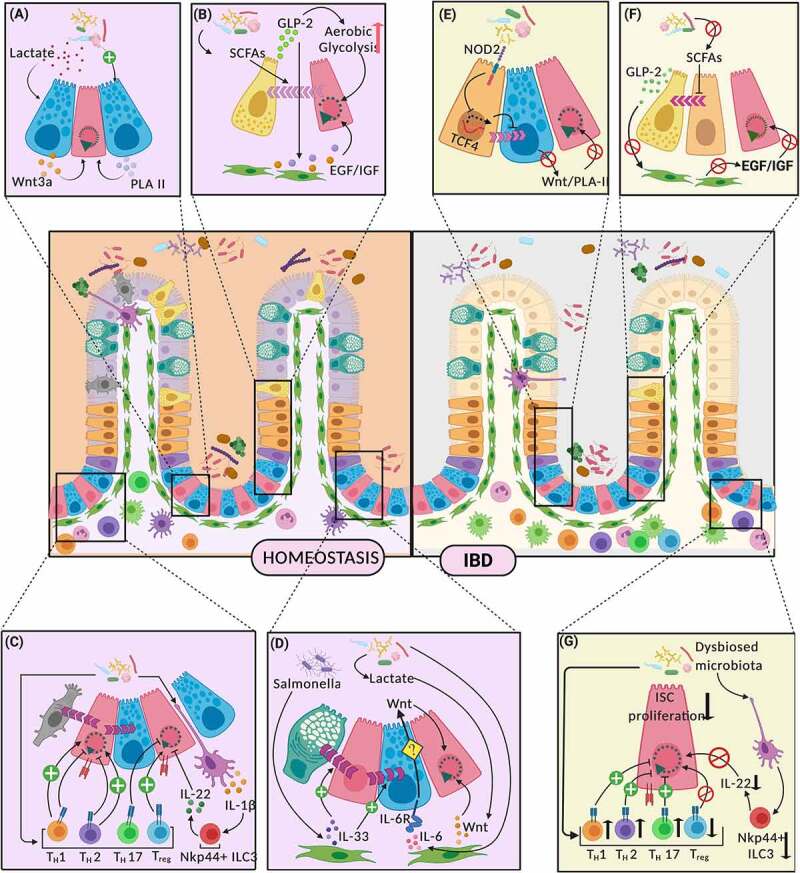
Indirect mechanisms of interaction between Gut Microbiota and LGR5+ Intestinal Stem Cells. (Figure 4 follows the key attached with Figure 3 for cell type reference) Significant interactions along the gut microbiota – intestinal stem cell axis, and their effects on proliferation and differentiation of ISCs have been depicted in homeostasis (left panel) and IBD (right panel). Paneth cells mediate these interactions through release of ISC proliferative signals like Wnt3A and phospholipase A2 (PLA II) (a). During IBD, these PC-mediated ISC-microbiota interactions are dysregulated (e). Enteroendocrine cells (EECs) forge microbiota-ISC interactions through the release of GLP-2 in response to microbial metabolites like SCFAs, which not only regulates their own differentiation from progenitors, but also drives underlying mesenchyme to release growth factors supporting ISC renewal (b). IBD – characterized dysbiosed microbiota leads to alterations in these interaction (f). Microbial stimulation can similarly lead to different helper T lymphocyte populations (Th1, Th2, Th17 and Treg) and ILC3, mediating either ISC self-renewal or differentiation (c). These mechanisms go haywire during IBD (g). Mesenchymal component of the ISC niche produces factors like IL-33, IL-6 and Wnt, in response to microbial signals, which regulate ISC proliferation and differentiation (d). PLA II (Phospholipase A2), SCFA (Short-chain fatty acids), GLP-2 (Glucagon-like peptide 2), EGF (Epidermal growth factor)/IGF (Insulin-like growth factor)

Paneth cells also possess exceptional plasticity, allowing them to dedifferentiate into ISC-like cells, in inflammatory conditions such as IBD. This capacity of Paneth cells aids in epithelial restitution and is known to be triggered by binding of stem cell factor (SCF) to a c-kit receptor on the Paneth cell surface.^72^ Interestingly, reports have shown SCF production by gut stromal and mast cells to be regulated by bacterial cues.^73,74^ Another important factor secreted by Paneth/ Paneth-like cells, which functions in regulation of intestinal stem cell homeostasis is the secretory phospholipase-II (PLA-2) enzyme. Gut inflammation promotes secretion of extracellular PLA2-IIA, by the Paneth cells, into the crypt lumen, relaying an autocrine signaling pathway, involved in production of prostaglandins.^75^ These prostaglandins can induce Wnt signaling, thereby supporting ISC proliferation and hence, salvage the epithelial layer during IBD. (Figure 4a and Figure 4e). Adding to its significance, PLA2-II activity is long known to be increased in the mucosa of patients with ulcerative colitis and Crohn’s disease.^76–78^ Interestingly, the expression of this secretory phospholipase has been shown to be regulated by bacterial components such as LPS, or bacteria-induced immune cytokines like IL-6, in multiple studies.^79–81^ In light of this knowledge, it would be an intriguing hypothesis to study if IBD-characteristic bacterial translocation across the mucosa, triggers an increased sPLA2 expression, in turn driving a restitutive ISC regeneration response.

Paneth cells are the functional foreground where IBD-associated genetic mutations (such as NOD2, ATG16L1, or XBP1) manifest their phenotypic consequences.^82–86^ Apart from allowing increased epithelial access to luminal pathogens, an altered Paneth cell functional profile also suggests a decrease in epithelial Wnt and Notch signaling, sided with a deficiency of growth factors required for stem cell proliferation and differentiation. Interestingly, CD is associated with decreased numbers or dysfunctional Paneth cells. The expression of TCF4, a target of the Wnt–β Catenin signaling that drives differentiation of ISCs toward Paneth cells, is reduced in ileal CD and has been traced back to a NOD2 mutation in Paneth cell progenitors. Since NOD2 is primarily a PAMP receptor, this observation hints at the involvement of the gut microbiota in this phenomenon.

### 4.2. Through Enteroendocrine cells

Enteroendocrine cells (EECs) form one of the largest endocrine organs in the human body. A subset of these cells, the L-cells, produce GLP-2, a molecule known to promote intestinal barrier integrity and crypt proliferation. One of the mechanisms by which the hormone supports epithelial proliferation is by enhancing aerobic glycolysis and channeling glycolytic substrates to amino acid biosynthesis in ISCs, thus emphasizing the role of metabolic reprogramming in intestinal homeostasis.^87^ Additionally, GLP-2 also acts as a niche signal by binding to GLP-2 receptors on subepithelial fibroblasts, where it induces expression of growth factors like EGF and IGF. These GFs in turn bind to and activate proliferating cells in the crypts^88,89^ (Figure 4b).

Intimate localization of EECs with the gut microbiota has been implicated in regulating the release of GLP-2. Multiple studies have demonstrated an increase in large intestinal tissue and serum levels of GLP-2, upon exposure to either purified bacterial short-chain fatty acids or to a combination of SCFA-producing bacteria and fiber-rich diet or prebiotics.^90–92^ The SCFAs bind to the FFA3 (or GPR41) receptor on the intestinal L-cells, signaling the release of GLP-2, thereby reducing inflammation, strengthening the epithelial tight junctions and stimulating epithelial proliferation.^91,93^ On the other end, gut microbiota also builds a link between the endocannabinoid system and intestinal epithelial homeostasis. Epithelial levels of endocannabinoid-like-bioactive-lipidsfor example, 2-oleoyl-glycerol (2-OG), have been reported to be modulated by bacteria such as *Akkermansia muciniphila*. These lipids act as epithelial gatekeepes and are known to act as ligands for GPR119, expressed on the surface of intestinal L-cells, where it promotes the release of GLP-2, eliciting anti-inflammatory effects.^94,95^

Significantly reduced levels of circulating and tissue-associated GLP-2 have been reported in IBD, while epithelial wound healing during restitution has been linked with improved GLP-2 levels.^96,97^ Given the evidence for regulation of GLP-2 expression via microbial cues, it might be postulated that the effects of the characteristic depletion of SCFAs in IBD may be mediated through reduced release of GLP2^98^ (figure 4f).

In the light of these observations, GLP-2 agonists have been studied as therapeutics in chemically or genetically induced murine IBD models and results are encouraging.^99^ Treatment with GLP-2 agonists had an anti-apoptotic and pro-proliferative action on crypt cells, resulting in increased crypt depth and villus height, decreased histological lesions, and increased intestinal weight and length. One GLP-2 agonist (teduglutide) is in clinical practice for treating short bowel syndrome.

## 5. ISCs Modulation by Gut Microbiota via the Immune Route

### 5.1. Through T-helper cells

The enriched CD4 + T lymphocyte population in the lamina propria, along with dendritic cells, macrophages, and innate lymphoid cells, are responsible for orchestrating the gut immune system in response to cues from gut commensals and dietary antigens. Gut microbiota regulates the gut T_H_ cell repertoire, controlling their maturation, differentiation, and plasticity.^100^ However, recent studies suggest that this is two-way traffic, wherein T helper cells interact with ISCs via MHC-II molecules expressed on the ISC surface. This interaction induces T_reg_ cells, a subclass of T_H_ cells, to produce IL-10 that promotes ISC self-renewal during homeostasis.^101^ Interestingly, IDO1, a kynurenine pathway enzyme, regulated by the gut microbiota, has been reported to support the expansion of gut Treg repertoire,^102^ further facilitating the gut microbiome–ISC interactions.

During bacterial and parasitic infections, Th1 and Th2 cell populations and their cytokines, inhibit ISC proliferation and drive their differentiation to Paneth and Tuft cells, respectively, to help contain infection. Similarly, TH17 cells have been shown to inhibit ISC proliferation, while encouraging differentiation.^101^

IBD is marked by aggressive and aberrant Th1 and Th17 responses to the dysbiotic gut flora. However, whether gut dysbiosis is the ‘cause’ or an ‘effect’ of this aberrant immune response remains elusive. Some studies of mucosal inflammation show altered gut oxygen levels and metabolic reprogramming, leading to expansion of facultative anaerobic and colitogenic microbes, supporting the hypothesis that gut dysbiosis is an ‘effect’ of altered niche environment in IBD.^103–105^ On the other hand, transfer of IBD-associated bacterial strains to gnotobiotic mice can elevate Th17 cells and their associated cytokines, thereby inducing gut inflammation.^106^ Irrespective of this ‘cause-effect dilemma’, it is evident that the gut microbiota-Th cells–ISC triad may go haywire in IBD, resulting in erratic gut Th-cell responses and promoting gut dysbiosis. (Figure 4c and Figure 4g)

### 5.2. Innate Lymphoid Cells

Innate Lymphoid Cells (ILCs) of the lamina propria represent a relatively new subset of regulators known to play a role in maintaining intestinal homeostasis. With their multifaceted roles in the eradication of infection, containment of microbiota, mitigation of inflammatory injury, and crosstalk with overlying epithelial cells, ILCs have managed to grab the attention of researchers. Lamina propria resident ILC3s and their cytokine IL-22 have been widely documented to maintain the ISC niche. Production of IL-22 from ILC3s is under the stimulation of microbiota-derived signals as evident from studies in gnotobiotic vs. CR mice. ILC3-derived IL-22 mediates STAT3 phosphorylation in ISCs and augments the growth of mice small intestine and human duodenal organoids.^107^ However, contrasting effects of IL-22 on ISCs and TA cells have been reported: IL-22 treatment leads to reduced Wnt /Notch signaling and decreased survival rates in ISCs, while TA cells are promoted to proliferate and differentiate. This confirms that gut microbiota–induced IL-22 production by ILC3s can either promote or impede epithelial regeneration, depending upon the target cell type in the crypt.^108,109^ Moreover, IL-22 protects ISCs against genotoxic stress, by activating DNA damage repair in ISCs, which initiates either quiescence or apoptosis, depending on the status of DNA repair.

Emphasizing their role in tissue repair and mucosal healing, IL-22 producing NKp44+ ILC3s appear important in IBD. Inflamed tissues in IBD show marked reduction in IL-22 producing NKp44+ ILC3s and enhanced IFN-gamma producing potential of the ILC3 pool.^110^ This IBD-associated shift in the ILC3 pool accounts for persistent inflammation with deteriorating epithelial barrier integrity and regeneration, through the lack of regulatory mechanisms listed above (Figure 4c and Figure 4g). The mechanisms triggering the protective role of ILC3s in the restitution of injury and inflammation remain elusive.

## 6. Gut microbiota–ISC crosstalk through stromal cells

Stromal cells, at one time termed the mesenchyme, are a conduit between luminal or epithelial signals and the submucosa. A dense network of stromal cells cups the intestinal epithelial layer, just beneath the basal membrane and is constantly in contact with epithelial cells, including ISCs. Stromal cell populations supplement the ISC niche with canonical and non-canonical Wnt ligands, R-Spondins, BMP inhibitors, PGE2, EGF and other essential growth factors required for sustenance of epithelial proliferation (details in Table 2). Although these extrinsic sources of Wnt and R-spondin works in synergy with epithelial Wnt, their ablation from murine models can lead to significant or complete loss of ISC proliferation, thus implying the vitality of stromal niche. Interestingly, fibroblast populations, surrounding the intestinal crypts, which are active producers of Wnt and BMP ligands, have been found to be dysregulated in both CD (SMA+ and Tenascin-C+ cells) and UC (CD142+ cells).^116,117^ Stromal niche members such as Gli1+ cells have also been implicated in epithelial restitution in colitis, as they are increased around the crypts after induction in murine DSS-colitis.^118^

**Table 2.** List of studies discussing the role of regulatory signals from the Mesenchymal Component of ISC niche on ISC proliferation and differentiation

**S**.**No**	**Mesenchymal Subpopulation**	**ISC niche factors produced**	**Biological Model**	**Observed effects on epithelial proliferation**
**1**	Whole stromal fraction, no subpopulation in particular^111^	Wnt(s) and RSPO3	Mice model with epithelial specific ablation Wnt signals (Porcn^Del^/ Villin Cre mice).	Stromal factors support epithelial proliferation and repair even in the absence of epithelial Wnts.
**2.**	CD34+ Gp38+ αSMA_ mesenchymal cells^112^	Wnt2b, Gremlin1, RSPO1	SI organoids cocultured with mesenchymal cells	Stromal factors enhance Wnt-β Catenin signaling in LGR5+ ISCs supporting epithelial proliferation.
**3.**	FoxL1+ telocytes^113^	Wnt2b, Wnt4, Wnt5a	FoxL1-hDTR mice administered with diphtheria toxin, leading to ablation of telocytes.	Reduced villus height, crypt depth; reduced stem cell proliferation (decreased Ki67 and Olfm4 expression); and reduced Wnts in subepithelial mesenchyme
**4.**	Extracellular vesicles (EV) derived from intestinal fibroblasts^114^	Amphiregulin (EGFR ligand)	Mice Small Intestinal and human colonic organoids treated with fibroblast-derived EVs	Treatment of organoids rescued them from the absence of EGF in the culture medium. The resultant increase in proliferation was seen to be mediated by EV-bound Amphiregulin, via activation of Wnt signaling in the ISC niche
**5.**	Gli1+ and ACTA2+ mesenchymal cells^115^	Wnt2b	Villin/Wls^cKO^ mice (with epithelium-specific knockout of Wingless) and intestinal organoids grown from crypts of these mice.	The knockout mice died within 14 days of the induction of mutation and showed altered villus morphology, absence of crypts in SI, and reduced expression of transcripts regulated by Wnt-β catenin signaling. Such mice and organoids were rescued by the supply of exogenous mesenchymal Wnt2b.

RSPO1, R-spondin 1; α-SMA, α-Smooth muscle actin (α-SMA); SI, Small Intestine; FoxL1-hDTR, BAC clone of human diphtheria toxin receptor gene driven by the Foxl1 promoter; EGFR, Epidermal Growth Factor Receptor; Gli1+, Glioma-associated oncogene homolog 1; ACTA2+, Actin Alpha 2.

Notably, intestinal fibroblasts express bacterial sensors such as TLRs 1–9 and NOD-1&2. Expression of these pattern recognition receptors, along with mediators of the NFk-B signaling pathway are upregulated after exposure to bacterial LPS or LTA.^119,120^ Stimulation of fibroblasts with live bacteria and their antigens also results in the production of active chemokines (CCL-2),^121,122^ cytokines (including IL-1α, 6,8) and growth factors (GM-CSF) by these cells.^123,124^ These mediators interact with surrounding cells to modulate mucosal immune responses and may regulate epithelial proliferation indirectly (Figure 4d).

Limited evidence also exists for microbial inducers of stromal signaling cascades, leading to ISC regeneration. Lactate derived from gut commensals can interact with pericryptal stromal cells and trigger the release of stromal Wnt signals, enhancing ISC proliferation^54^ (Figure 4a). *Salmonella* infection can also trigger the pericryptal myofibroblasts to express IL-33, which can program ISC and progenitor cell differentiation toward a secretory phenotype, via ST2-dependent signaling. As a consequence, an increased number of Paneth and ckit+ goblet cells not only mount an antimicrobial defense, but also increase epithelial turnover by supplying Wnt ligands.^124^ Myofibroblast-derived IL-6 might also be a lead to follow, because it is known to be produced in response to microbial exposure and finds its receptors (IL6Rs) on proximally located Paneth cells. Stimulation of Paneth IL-6Rs with epithelial-derived IL-6 is known to switch the STAT3 signaling ON, promoting secretion of ISC proliferative factors by small intestinal Paneth cells. (Figure 4d) Whether stromal IL-6 can elicit a similar response is an intriguing question.^125^ Even though Paneth cells are missing in normal colonic crypts, IL-6 is also known to induce colonic epithelial proliferation, although the mechanism is unclear. IL-6 mediated proliferation and restitution in colonic mucosa is consistent with IL-6 accumulation around colonic perforation.^126^ The gut microbiota–mesenchyme–ISC axis still leaves a lot to be explored and understood. Strategic positioning of pericryptal stromal cells and their evident cross-talk with immune and epithelial cells during microbial exposure and epithelial barrier breach warrant extensive investigation.

## 7. Conclusions

IBD is the cumulative consequence of a disturbed gut microbiota in a genetically predisposed environment, with an under-regulated, heightened immune reaction. Marked distortions in the gut epithelium cellular structure and functions have been attributed to IBD. As the ISCs are the chieftains of epithelial renewal and differentiation, further studies investigating the role of the ISC and its niche in gut inflammatory disorders, are warranted.

As the dysbiotic gut microbiome acts as a primary ‘ON’ switch for the inflammatory circuit in IBD, IBD therapeutics have largely focused on targeting the inflammatory immune pathways and restoration of the gut microbiome. However, with the advent of more sophisticated model systems such as the organoids, the focus of the translational IBD research has shifted to regenerative medicine. Autologous organoid transplantation experiments and their success in restoration of healthy gut epithelium in mice models may unfurl newer avenues in regenerative medicine for targeting IBD.^127,128^ The therapeutic potential of the ISC transplantation relies heavily on our understanding of the ISC niche during health and disease.

IBD-associated rewiring of the ISC niche has been established as a key event in the IBD pathophysiology. Understanding of specific microbial mechanisms regulating the ISC niche and their alterations during IBD shall pave way to design more holistic therapeutics, which may enhance the efficacy of the microbiome restoration therapies that are currently being utilized or are being actively researched (fecal microbiota transplantation, probiotics, synbiotics, postbiotics, etc.) to curb IBD.

## Data Availability

Data sharing is not applicable to this article as no datasets were generated or analyzed during the current study.
